# Transcranial direct current stimulation (tDCS) over the orbitofrontal cortex reduces delay discounting

**DOI:** 10.3389/fnbeh.2023.1239463

**Published:** 2023-08-24

**Authors:** Andrea Stefano Moro, Daniele Saccenti, Alessandra Vergallito, Simona Scaini, Antonio Malgaroli, Mattia Ferro, Jacopo Lamanna

**Affiliations:** ^1^Department of Psychology, Sigmund Freud University, Milan, Italy; ^2^Center for Behavioral Neuroscience and Communication (BNC), Vita-Salute San Raffaele University, Milan, Italy; ^3^Transcranial Magnetic Stimulation Unit, Italian Psychotherapy Clinics, Milan, Italy; ^4^Department of Psychology and NeuroMi, University of Milano-Bicocca, Milan, Italy; ^5^Child and Youth Lab, Sigmund Freud University, Milan, Italy; ^6^Child and Adolescent Unit, Italian Psychotherapy Clinics, Milan, Italy; ^7^Faculty of Psychology, Vita-Salute San Raffaele University, Milan, Italy; ^8^San Raffaele Turro, IRCCS Ospedale San Raffaele, Milan, Italy

**Keywords:** delay discounting, neuromodulation, transcranial direct current stimulation, orbitofrontal cortex, decision-making, prefrontal cortex, intertemporal choice

## Abstract

Delay discounting (DD) is a quantifiable psychological phenomenon that regulates decision-making. Nevertheless, the neural substrates of DD and its relationship with other cognitive domains are not well understood. The orbitofrontal cortex (OFC) is a potential candidate for supporting the expression of DD, but due to its wide involvement in several psychological functions and neural networks, its central role remains elusive. In this study, healthy subjects underwent transcranial direct current stimulation (tDCS) while performing an intertemporal choice task for the quantification of DD and a working memory task. To selectively engage the OFC, two electrode configurations have been tested, namely, anodal Fp1–cathodal Fp2 and cathodal Fp1–anodal Fp2. Our results show that stimulation of the OFC reduces DD, independently from electrode configuration. In addition, no relationship was found between DD measures and either working memory performance or baseline impulsivity assessed through established tests. Our work will direct future investigations aimed at unveiling the specific neural mechanisms underlying the involvement of the OFC in DD, and at testing the efficacy of OFC tDCS in reducing DD in psychological conditions where this phenomenon has been strongly implicated, such as addiction and eating disorders.

## 1. Introduction

Frequently, people encounter circumstances in which they must decide between immediate and future gains. Such decisions necessitate the cognitive representation of forthcoming rewards, alongside an assessment of their subjective value, which is performed by taking into account several factors, including the rewards’ magnitude, as well as the effort and the waiting time required to receive them (Frost and McNaughton, 2017). In this context, the subjective value of a reward diminishes with the delay to its receipt, a phenomenon referred to as delay discounting (DD) (Amlung et al., 2019).

Although the phenomenology of DD has been deeply explored, suggesting that it is a universal, cognitive mechanism with comparable characteristics among many animal species, only a few experimental studies in humans investigated whether a specific brain circuit supporting DD exists (Frost and McNaughton, 2017). From a theoretical perspective, McClure et al. (2004) speculated the existence of two interacting systems underlying the DD process, named β and δ, respectively. The former accounted for the “immediacy” nature of the decision outcome, while the latter related to “all decisions” (McClure et al., 2004). In contrast, Kable and Glimcher (2007) proposed the notion of a unique system widespread in the brain that computes the subjective value of heterogenous rewards. A further hypothesis was advanced by Hare et al. (2009), who asserted that goal-directed decisions have their roots in a common value signal encoded in the ventromedial portion of the frontal cortex, whereas exerting self-control involves the modulation of such signal by the dorsolateral prefrontal cortex (DLPFC).

However, it is also truth that subjects with a lesion in the ventromedial cortex displayed unstable but transitive preferences during decision-making (Yu et al., 2022). At a glance, currently available studies indicate the role of some candidate brain regions, but results are still controversial and one cannot exclude the fact that DD is processed in a unique brain area *a priori* (Moro et al., 2023). In addition, the interactions between DD and other domains of executive functioning remain elusive. As an example, it is widely acknowledged that working memory (WM) plays a role in DD (Bickel et al., 2011; Szuhany et al., 2018), but the nature of such a relationship is debated since aging-dependent WM decline does not explain the enhanced delayed gratification which is observed in older subjects (Hernandez et al., 2017). Furthermore, WM training does not always affect discounting rates significantly (Renda et al., 2015). Considering the devaluation of rewards as a function rooted in choice impulsivity (Odum, 2011), another issue concerns the absence of a direct relationship between DD and general impulsivity-related constructs. As a matter of fact, albeit experimental studies highlighted that higher ratings of overall trait impulsivity, non-planning impulsivity, and motor impulsivity are associated with greater DD measures (de Wit et al., 2007; Perales et al., 2009; Lewis et al., 2015; Jauregi et al., 2018), another evidence contradicts this idea (Caswell et al., 2015). To fill these gaps, a valuable approach would be to stimulate the candidate’s brain area while the subject performs both an intertemporal choice task and other cognitive tests assessing performance in other domains. To this aim, non-invasive neural stimulation (NIBS) represents an invaluable means for investigating the functional role of specific brain areas and circuits thought to be involved in psychological processes (Ferro et al., 2022; Moro et al., 2023). Among NIBS techniques, transcranial direct current stimulation (tDCS) represents a well-tolerated non-pharmacological tool to modulate the membrane potentials of cortical neurons, thus influencing their excitability, spontaneous and evoked firing rates, and activity synchronization (Nitsche et al., 2008). Moreover, both voltage and electric fields generated by tDCS can be accurately simulated through mathematical models and dedicated software (Nasimova and Huang, 2022).

The dorsolateral prefrontal cortex (DLPFC) is one of the most widely implicated brain regions in the context of DD, as indicated by several studies. In general, the cathodal stimulation of both the left and right DLPFC is known to produce a reduction in DD (Hecht et al., 2013; He et al., 2016; Shen et al., 2016; Kekic et al., 2017; Nejati et al., 2018; Xiong et al., 2019; Brunelin and Fecteau, 2021). However, it is important to note that there are other brain regions that have not been thoroughly investigated yet and may also play a role in DD. Undoubtedly, the orbitofrontal cortex (OFC) is among the most promising candidate brain regions that might play a central role in DD, as highlighted by our recent literature analysis (Moro et al., 2023). Consistent with this hypothesis, Sellitto et al. (2010) have also shown that injuries to the medial aspect of OFC result in steeper DD of future rewards.

The OFC has been more specifically associated with “hot” executive functions, i.e., those higher-order psychological processes related to motivation and emotion, compared to DLPFC. The latter is thought to be mostly involved in “cold,” purely cognitive, executive processes (Nejati et al., 2018), although functional changes of its circuitry, both internal and external, have been related to stress-related responses (Cerqueira et al., 2007; Lamanna et al., 2020) and depressive-like behaviors (Biselli et al., 2021; Lamanna et al., 2022).

Turning to animal models with lesions to the OFC, currently available studies emphasized the significance of this structure in DD expression (Mar et al., 2011; Jo et al., 2013). More specifically, lesions of the OFC promote a preference for smaller, immediate reinforcements over larger, delayed ones (Mobini et al., 2002). However, OFC damages can also produce opposite effects, i.e., an increasing preference for larger but delayed rewards (Winstanley et al., 2004), suggesting that OFC may contribute to consistent, transitive decision-making (Kable and Glimcher, 2010). OFC is also anatomically linked to multiple brain areas involved in decision-making, such as the ventral tegmental area, nucleus accumbens, dorsal raphe, amygdala, and hippocampus (Ballard and Knutson, 2009; Peters, 2011; Stalnaker et al., 2015). Moreover, the OFC is notably vulnerable to reductions in gray matter volume resulting from aging, which could explain why older individuals frequently choose larger, delayed rewards over smaller, immediate ones (McNamara et al., 2008). Significant alterations in OFC white matter volume have been observed in patients suffering from anorexia nervosa, a clinical population known to exhibit extremely low degrees of DD (Shott et al., 2016; Lamanna et al., 2019; Spadini et al., 2021).

Despite the importance of the subject’s matter, only a limited number of empirical studies have explored the impact of OFC neurostimulation on intertemporal choice dynamics in humans. With this regard, Nejati et al. (2018) targeted the OFC using NIBS during a DD task and observed a decrease in devaluation rates. However, it should be noted that in this study, the reference electrode for tDCS was placed on the DLPFC, thus indicating that this brain region was also inevitably stimulated during the experiment (Nejati et al., 2018). More recently, Manuel et al. (2019) applied tDCS over the ventromedial portion of the frontal cortex and demonstrated an effect of stimulation on DD.

On these bases, the aim of our experimental work was to design a tDCS configuration able to stimulate the OFC more focally, and to investigate its involvement in DD. At the same time, we evaluated if the subject’s performance in the intertemporal choice task depended on other cognitive functioning domains, namely, general impulsivity and WM.

## 2. Materials and methods

### 2.1. Participants

Forty-two participants, consisting of 11 males and 31 females with a mean age of 24 ± 2.62 years, were equally divided into two cohorts. Most of the participants (*n* = 25) held a bachelor’s degree, while 11 participants had a master’s degree. None of the participants reported any history of psychiatric or neurological disorders, nor had contraindications to tDCS (Antal et al., 2017). Recruitment for this study was conducted on a word-of-mouth basis at a university site. Participants’ ethical treatment was in accordance with the principles stated in the Declaration of Helsinki and was approved by the Ethics Board of the Faculty of Psychotherapy Science and the Faculty of Psychology of Sigmund Freud University (Vienna, Austria; protocol number: JBWXE8CIAVWD788416). Informed consent was obtained from all subjects.

### 2.2. Transcranial direct current stimulation

**FIGURE 1:**
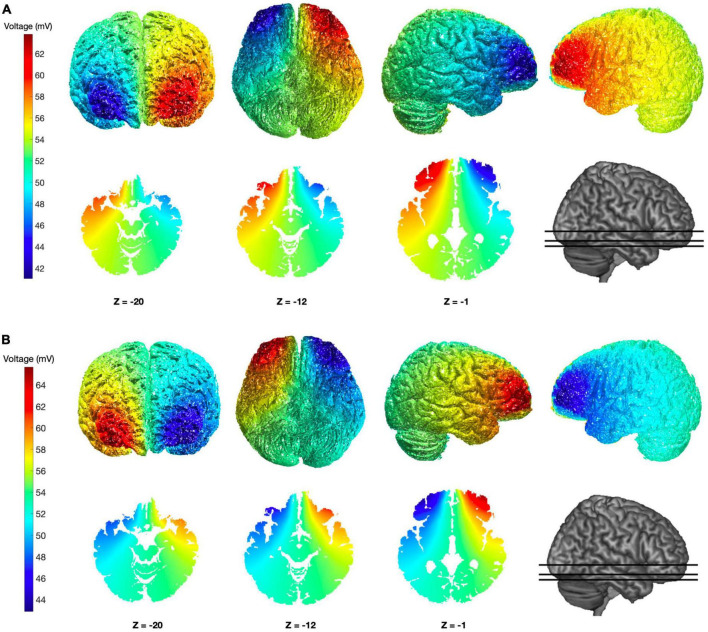
Simulation of brain electric potential distributions for the tDCS configurations adopted. **(A,B)** Colormap images show the putative distribution of the electric potential obtained using the custom electrodes configuration adopted, at both cortical and subcortical levels and with different view-points, based on a computational simulation performed with ROAST (Huang et al., 2019). The simulation was conducted using the “MNI152_T1_1 mm” template and assuming current intensity of 2 mA through two bilateral electrodes configurations: anodal Fp1–cathodal Fp2 **(A)** and cathodal Fp1–anodal Fp2 **(B)**.

The tDCS current stimulus consisted of 2 mA DC delivered using a BrainSTIM device (E.M.S. s.r.l., Italy) to the Fp1 and Fp2 sites (10–20 EEG system) with 5 × 5 cm^2^ electrodes, resulting in a current density of 0.08 mA/cm^2^. As illustrated in Figure 1, two stimulation polarities and a Sham (no stimulation) condition were adopted: anodal Fp1–cathodal Fp2 (also referred to as Anodal Left/Cathodal Right in the following); cathodal Fp1–anodal Fp2 (Anodal Right/Cathodal Left) (Figure 1). The current stimulus was continuously delivered for 20 min, including two ramping periods of 30 s at both the beginning and the end of stimulation. On the contrary, in the Sham condition, no stimulation was provided, with the exclusion of a sequence of two opposite ramping stimuli, each lasting 15 s, where the current was raised from 0 to 2 mA and immediately lowered from 2 to 0 mA, that was delivered both at the beginning and at the end of a 20 min time window, i.e., the stimulation period of the other conditions.

### 2.3. Psychological measures

Each participant underwent testing using the Italian version of the Barratt Impulsiveness Scale-11 (BIS-11) (Fossati et al., 2001) which assesses global, motor (BIS_M_), non-planning (BIS_N_), and attentional impulsivity (BIS_I_). Behavioral impulsivity was assessed through a computerized Go/NoGo task created using PEBL 2.1 software (Mueller and Piper, 2014). The stimuli for this task were the letters “P” and “R” presented randomly in one of four square grids with a blue star in the middle. The stimulus time was 500 ms followed by a blue star for 500 ms, resulting in an interstimulus time of 1,000 ms. The task comprised 480 Go stimuli (80%) and 120 NoGo stimuli (20%), presented in two separate blocks of 300 stimuli each, with a short break between the blocks and the “P” and “R” letters inverted (i.e., “P” become “R” and vice versa) in the second block. On average, the Go/NoGo task required approximately 20 min. For this reason, it was not feasible to administer it during tDCS stimulation. DD was evaluated using a computerized custom-made monetary intertemporal choice task (MICT) developed in jsPsych^1^ that determined the subjective value/indifference point for six temporal delays (1, 6, 12, 24, 60, and 120 months) for two maximal reward values: 500 € and 10,000 €, using the adjusting immediate amount procedure described by Holt et al. (2012). Participants had to choose between an immediate but adjusted reward or a maximal but delayed one during each step of the task. Briefly, the test involves making a choice between the full reward (i.e., 500 or 10,000 €) at one of the previously indicated delays (1, 6, 12, 24, 60, and 120 months) or receiving half of that value immediately. Depending on the choice, the immediate reward is increased by half of the difference between its current amount and the maximal one if the subject chooses the delayed reward, or it is reduced by the same amount if the subject chooses the immediate one. This approximation process is repeated for 6 rounds, and at the end, the IP for that specific reward and delay is determined. The reward and delay are presented randomly.

Working memory performance was assessed using a computerized n-Back task created with PEBL 2.1 software (Mueller and Piper, 2014). The task consisted of two blocks, 2nback and 3nback, each comprising 32 and 33 letters/items presented at intervals of 1,500 ms. Participants received brief training for each task before starting the experiment.

### 2.4. Experimental design

A double-blind between-subject design was adopted for this study, wherein participants underwent two sessions spaced at least 1 week apart, regardless of their random cohort assignment. During the first session, participants completed the BIS-11 and Go/NoGo tasks. Afterward, they underwent the first tDCS stimulation, which was randomly assigned as either real or sham. Six minutes after the stimulation began, participants were tested with the MICT and n-Back tests in random order. During the second session, participants only underwent the stimulation protocol (real or sham) with the MICT and n-Back tests. For the first cohort of subjects (*n* = 21), the tDCS anodal electrode was placed over Fp1 while the cathodal electrode over Fp2, whereas for the second cohort (*n* = 21), the anodal electrode was placed over Fp2 while the cathodal electrode over Fp1.

### 2.5. Statistical analysis

For DD analysis, indifference points (IPs) from each subject/session obtained with the MICT were used to fit, for each subject, two hyperbolic function IP = A/(1 + k ⋅ Delay) (Mazur, 1987), one when A = 10,000 € and the other one 500 € depending on subtask and Delay = 1, 6, 12, 24, 60, and 120 months. By analyzing the test execution times, we observed that subjects took an average of 231.7 ± 64.9 s (mean ± std). Then, we use the non-linear least-mean squares method to get estimates of the devaluation coefficient k (with unit 1/days), while the areas under the curve (AuC) were calculated and normalized following the procedure described by Myerson et al. (2001): the delay was expressed as a proportion of the maximum delay, while the subjective value was expressed as a proportion of the nominal amount, i.e., the subjective value divided by the actual, delayed amount. We fitted multiple linear mixed-effect models (LMEs) to our data, including the logarithm of k and the AuC as dependent variables and different fixed and random effect factors as detailed in the results section, followed by ANOVA or Kenward–Roger tests on the fitted models. The normality of AuC and log(k) distributions, as well as of the LME residuals, were evaluated graphically using qqplots and histograms. All measures obtained from tests in all stimulation conditions were evaluated for the presence of significant correlation using Pearson’s correlation coefficients, corrected using Holm–Bonferroni method. To compare the scores of n-Back performances, Wilcoxon signed-rank tests were conducted. All the analyses were carried out using custom algorithms developed in MATLAB (Mathworks) or R.^2^

## 3. Results

### 3.1. Transcranial direct current stimulation and delay discounting

The main aim of this study was to evaluate if OFC neural activity is critically involved in the expression of DD observed with intertemporal choice tasks. To this aim, we opted for testing the effects of tDCS delivered over the OFC on DD performance. Since focusing tDCS stimulation on this brain area is challenging, we evaluated several possible electrode configurations starting from those already adopted by other investigators. Both electric field and electric potential distributions were simulated using the ROAST^3^ MATLAB package (Huang et al., 2019). Finally, we opted for a custom configuration aiming at bilateral interhemispheric stimulation of OFC. For our configuration, electrodes are placed on Fp1 and Fp2 sites and both current directions were tested in separate subjects (see methods for details): putative electric potential profiles for the two polarities are shown in Figure 1, over the cortical surface as well as over three horizontal brain sections including subcortical regions. As it can be appreciated by looking at the colormaps, sensible stimulation intensities are reached over the medial and lateral portions of the OFC of both hemispheres (with reversed voltage polarities); at the same time, the spread of stimulation is noticeably reduced over the DLPFC and ventromedial prefrontal cortexes, while the frontopolar cortex is inevitably involved (Figure 1).

We then recruited two cohorts of healthy subjects for testing the effects of the two stimulus configurations (compared to the sham condition) on their performance in the MICT and the n-Back tasks (see the section “2. Materials and methods”).

**FIGURE 2:**
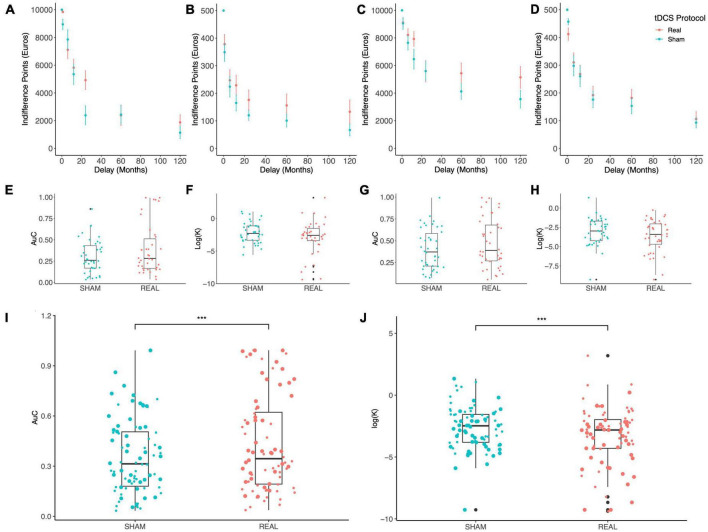
Results of the monetary intertemporal choice task. **(A–D)** The indifference points obtained at each delay are shown for the Sham (blue) and Real (red) tDCS with: Anodal Left/Cathodal Right electrodes configuration and 10,000 € **(A)** or 500 € **(B)** maximal reward; Anodal Right/Cathodal Left configuration and 10,000 € **(C)** or 500 € **(D)** maximal reward. **(E–H)** Box plots display the delay discounting measures Area under the Curve (AuC) and the logarithm of the discounting rate k in the Anodal Left/Cathodal Right **(E,F)** and Anodal Right/Cathodal Left **(G,H)** electrodes configurations. **(I,J)** Lastly, box plots show pooled data for AuC **(I)** and the logarithm of k **(J)**, where big and small dots indicate the 10,000 € and 500 € maximal reward conditions, respectively; tDCS significantly increased AuC [Real vs. Sham: *F*(1,154) = 11.723, *p* = 0.0008; main effect of reward: *F*(1,154) = 39.117, *p* < 10^– 5^; ANOVA on linear mixed-effects model] and reduced k [Real vs. Sham: *F*(1,154) = 13.453, *p* = 0.0003; main effect of reward: *F*(1,154) = 44.036, *p* < 10^– 5^; ANOVA on linear mixed-effects model]. ***p < 0.001.

Figure 2 shows the indifference points at varying delays which were obtained with the MICT under Sham (blue) vs. Real (red) stimulation: a clear trend can be appreciated where IPs are increased by both stimulation polarities (Anodal Left, Figures 2A, B; Anodal Right, Figures 2C, D), for both maximal reward levels (10,000 €, Figures 2A, C; 500 €, Figures 2B, D), thus indicating a reduction of delay discounting by OFC tDCS stimulation. To evaluate if such an effect was significant, we used two different measures of DD, the devaluation coefficient k from the hyperbolic fitting of IPs and the AuC (see the section “2. Materials and methods”). Due to the skewness of k distributions, we log-transformed this coefficient as suggested previously (Holt et al., 2012). Values of k and AuC are separately shown in Figure 2 for the two stimulation polarities (Anodal Left, Figures 2E, F; Anodal Right, Figures 2G, H), while pooled data are shown in Figures 2I, J. An average trend can be observed where AuC is increased by the stimulation while log(k) is reduced, thus indicating a possible reduction of discounting exerted by tDCS. Two different LME models were fitted to these data, according to the following formulas (Wilkinson notation):


(1) y∼1+Real⋅Polarity⋅Reward+TestOrder⋅Real

+StimulationOrder⋅Real+BIS-11+Gonogo

+Nback2+Nback3+(1|subject)

And


(2) y∼1+Real⋅Polarity⋅Reward+Test⁢Order⋅Real

+Stimulation⁢Order⋅Real+(1|subject),

Where y is the response variable (log(k) or AuC). Among the fixed effect factors evaluated, *Real* indicates whether stimulation is Real or Sham, *Polarity* indicates the polarity of stimulation, *Reward* indicates the maximal reward of the MICT, i.e., if the curve were calculated when the delayed reward was € 10,000 or € 500 (all interactions terms have been included for these three factors), while *BIS-11*, *Nback2*, and *Nback3* indicate the performances in the related tasks. *Test Order* and *Stimulation Order* indicate the task presentation order and effect of session order, respectively. Finally, a random effect of the subject was included.

Comparison of the two models using the Kenward-Roger test showed no significant difference for either log(k) (χ^2^_4_ = 3.6304, *p* = 0.4583) or AuC (χ^2^_4_ = 5.7006, *p* = 0.2226), thus excluding a significant contribution of either general impulsivity or WM performance on subject’s DD performance in our conditions. Furthermore, we compared Equation 2 model with a more parsimonious one that did not include factors related to the order of tests and stimulation:


(3) y∼1+Real⋅Polarity⋅Reward+(1|subject),

Comparison of the two models using the Kenward-Roger test showed no significant difference for either log(k) (χ^2^_4_ = 7.9083, *p* = 0.095) or AuC (χ^2^_4_ = 8.4905, *p* = 0.07517). Based on this, we performed our analyses on the more parsimonious model of equation 3.

Analysis of variance applied to the fitted models revealed a significant effect of tDCS stimulation and maximal reward of the MICT [tDCS: *F*(1,154) = 13.453, *p* = 0.0003; Reward: *F*(1,154) = 44.036, *p* < 10^–5^; Figure 2I]. Interestingly, no significant effect of electrodes configuration (i.e., stimulus polarity, *Polarity*) was found [for log(k): *F*(1,38) = 2.1052, *p* = 0.1547932; for AuC: *F*(1,38) = 1.9633, *p* = 0.169071], as well as no significant interaction between pairs of factors. Importantly, the same results were obtained when AuC was set as the response variable [tDCS: *F*(1,154) = 11.723, *p* = 0.0008; Reward: *F*(1,154) = 39.117, *p* < 10^–5^; Figure 2I].

Stimulation of this brain region with two different tDCS configurations revealed a general effect implicating the OFC in this decision-making process. Based on these results, we can conclude that bilateral tDCS focused over the OFC seems to reduce DD in healthy subjects, independently from stimulus polarity. Graphically, it appears that stimulation was more effective for specific delays which is consistent with the notion that intertemporal choice involves different brain circuits depending on the reward delay (Wittmann et al., 2007; Frost and McNaughton, 2017).

### 3.2. Impulsivity and delay discounting

**FIGURE 3:**
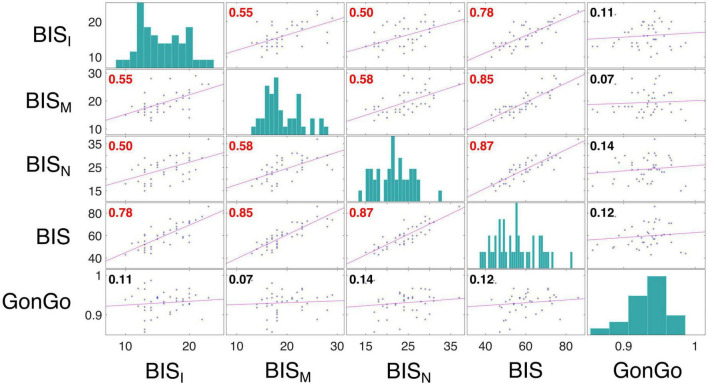
Correlation analysis among impulsivity domains. Correlation analysis was performed between measures from the BIS scores, including the dimensions BIS_I_, BIS_M_, and BIS_N_, and Go/NoGo task accuracy (GonGo) (data from both groups were pooled). Each off diagonal square contains a scatterplot of a pair of variables with a least-squares reference line, the slope of which is equal to the displayed correlation coefficient (indicated by the numerical value in the upper left corner; red font color indicates statistical significance corrected with Holm-Bonferroni method for the variable presented in the matrix, *p* < 0.05). Each square on the diagonal shows the histogram of the measure. The correlation between BIS dimensions were: BIS_I_ and BIS_M_, *r* = 0.55, *p* = 0.0009; BIS_I_ and BIS_N_, *r* = 0.50, *p* = 0.0034; BIS_I_ and BIS, *r* = 0.78, *p* < 0.0001; BIS_M_ and BIS_N_, *r* = 0.58, *p* = 0.0003; BIS_M_ and BIS, *r* = 0.85, *p* < 0.0001; BIS_N_ and BIS, *r* = 0.87, *p* < 0.0001.

**FIGURE 4:**
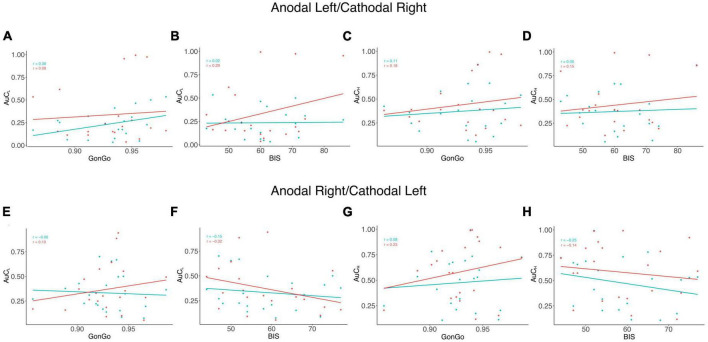
Correlation analysis among impulsivity domains and DD. Correlation analysis was performed between measures from the area under the curve (AuC) values of DD experiments with 500 € (AuC_L_) and Go/NoGo task accuracy (GonGo) **(A)** and the global BIS scores (BIS) **(B)**, the area under the curve of 10,000 € (AuC_H_) with GonGo **(C)**, and BIS **(D)** in the Anodal Left/Cathodal Right condition. In the lower part of the figure, there were correlations between AuC_L_ and GonGo **(E)** and BIS **(F)** and between AuC_H_ and GonGo **(G)** and BIS **(H)** in the Anodal Right/Cathodal Left condition. The blue dots and lines represent the SHAM condition, while the actual stimulation situation is represented in red. None of the observed correlations were significant.

The LME analysis presented here contradicts the idea that a clear link exists between impulsive behavior and DD (Moreira and Barbosa, 2019), as none of these variables significantly contributed to the model fitting for either the AuC or log(k). Moreover, all the correlations between the impulsivity traits, BIS-11, and behavioral impulsivity, Go/NoGo, were not significant. On the contrary, as expected, the internal sub-dimension (BIS_M,_ BIS_N_, and BIS_I_) are correlated with each other and with the overall score (BIS) (Figure 3). To better evaluate this aspect, as well as the relationship between DD performance and all the other impulsivity measures, we conducted a correlation analysis separately for the three stimulation conditions: Sham, Anodal Left/Cathodal Right, and Anodal Right/Cathodal Left. From these analyses, no significant correlations were found between either impulsivity personality traits (BIS-11) or motor impulsivity (Go/NoGo), both assessed before experiments, and intertemporal choice performance (AuC) measured during any experimental condition (Figure 4). Therefore, we can conclude that DD expression in our condition can be considered as a distinct psychological process that is not merely a manifestation of impulsivity.

### 3.3. Working memory and delay discounting

**FIGURE 5:**
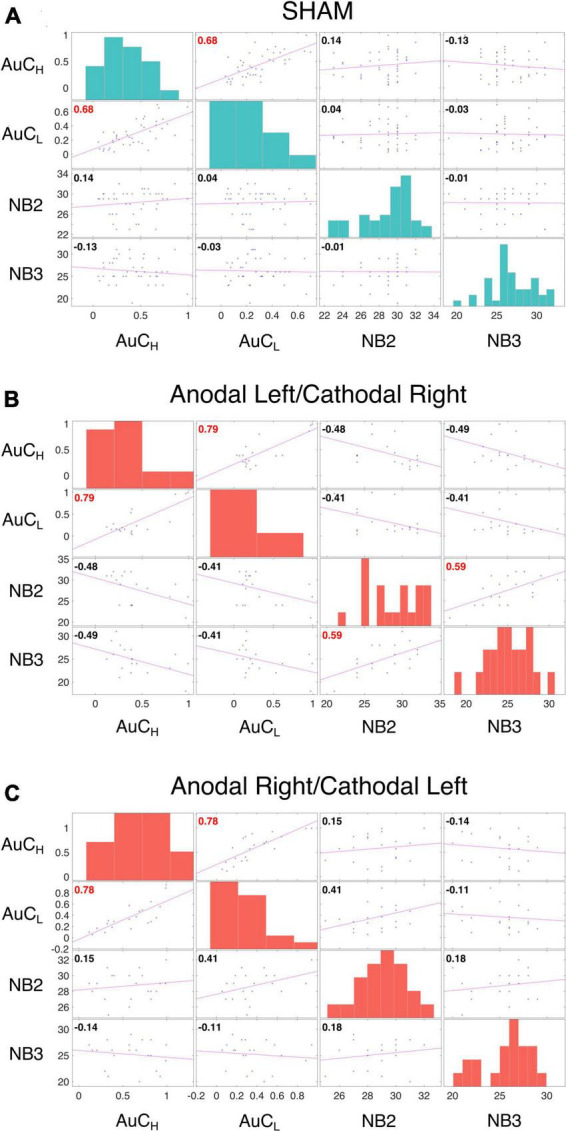
Correlation analysis of DD and WM. Correlation analysis was performed between measures from the area under the curve (AuC) values of DD experiments with 10,000 € (AuC_H_) and 500 € (AuC_L_), and the n-Back task performance with two (NB2) and three (NB3) letters for participants who received SHAM (data from both groups were pooled, **(A)**, Anodal Left/Cathodal Right stimulation **(B)** and Anodal Right/Cathodal Left stimulation **(C)**. Each off diagonal square contains a scatterplot of a pair of variables with a least-squares reference line, the slope of which is equal to the displayed correlation coefficient (indicated by the numerical value in the upper left corner; red font color indicates statistical significance corrected with Holm-Bonferroni method separately for the variable presented in each matrix, *p* < 0.05). Each square on the diagonal shows the histogram of the measure. The Performance of DD revealed a significant correlation in all three conditions between 500 € and 10,000 € (SHAM: AuC_H_ and AuC_L_: *r* = 0.68, *p* < 0.0001; Anodal Left/Cathodal Right: *r* = 0.79, *p* = 0.0004; Anodal Right/Cathodal Left: *r* = 0.78, *p* = 0.0002). While the n-Back performances showed a significant correlation only in the Anodal Left/Cathodal Right stimulation (NB2 and NB3: *r* = 0.59, *p* = 0.0083), and a significant difference in correlations between the real and SHAM conditions for the same electrodes configuration (Pearson and Filon’s *z*-test: *z* = 2.9385, *p* = 0.003).

**FIGURE 6:**
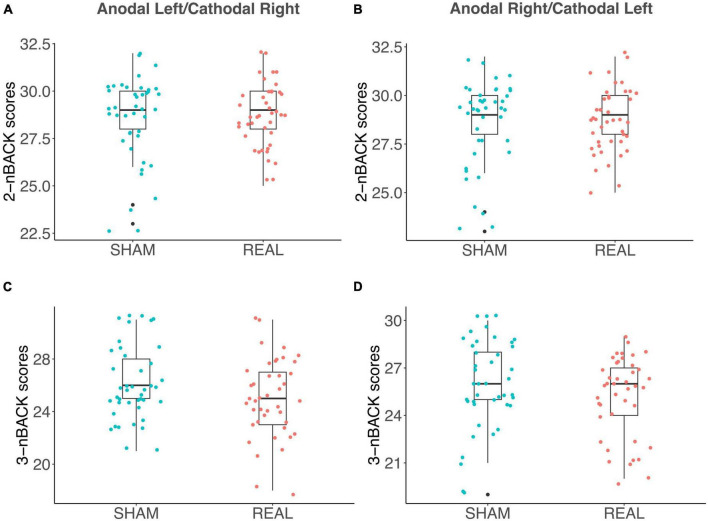
Effects of tDCS on the n-Back tests for working memory. **(A–D)** Box plots show n-Back task performance (i.e., the number of correct letters, identified by the participants) in the Anodal Left/Cathodal Right stimulation configuration for both 2-letter **(A)** and 3-letter **(C)** task’s variants, as well as the n-Back task performance in the Anodal Right/Cathodal Left stimulation condition for both 2-letter **(B)** and 3-letter **(D)** conditions. Blue and red dots indicate Sham and Real tDCS stimulation, respectively. No significant differences were found between Sham and Real conditions for any comparison (Wilcoxon signed-rank test, two-tails, n.s.).

As discussed in the introduction, the relationship between WM and DD is controversial (Moro et al., 2023) and WM and DD are typically positively correlated (Wesley and Bickel, 2014). We did not detect a significant contribution of WM performance in fitting LMEs models to either log(k) or AuC data. We also observed no significant correlation between n-Back performance and the AuC of DD in Sham (Figure 5A), in the Anodal Left/Cathodal Right stimulation condition (Figure 5B), or in the Anodal Right/Cathodal Left stimulation condition (Figure 5C): these results confirmed our LME analysis. However, in the Anodal Left/Cathodal Right stimulation condition, we found a significant positive correlation between the n-Back performances (nback2 and nback3: *r* = 0.59, *p* = 0.0083, statistical significance corrected with Holm-Bonferroni method; Figure 5B). We also conducted tests to compare correlations using correlation comparison tests (Diedenhofen and Musch, 2015) and found that the correlation in the Anodal Left/Cathodal Right configuration was significantly different from the one obtained in the related Sham condition. More specifically, our results showed a significant difference using Pearson and Filon’s *z*-test (*z* = 2.938, *p* = 0.003). Altogether, such results might reveal a potential specific modulatory action of OFC on WM independently of intertemporal choice processes. To further evaluate this aspect, we tested whether tDCS altered the WM performance as assessed through the n-Back tasks, whose results are shown in Figure 6 for the two stimulation polarities (Anodal Left, Figures 6A, C; Anodal Right, Figures 6B, D). Both 2nback and 3nback performances did not differ significantly between the Real and Sham conditions (Wilcoxon signed-rank test, two-tails, n.s.; Figure 6).

## 4. Discussion

Although several authors have previously discussed the involvement of the OFC in intertemporal choice tasks (Kable and Glimcher, 2007; McClure et al., 2007; Schoenbaum and Shaham, 2008; Peters and Büchel, 2010; Miyazaki et al., 2020), to our knowledge this is the first study to investigate the engagement of the OFC in DD using tDCS with minimal involvement of other cortical areas, such as the dorsolateral prefrontal cortex. Our optimized tDCS configuration, designed based on electric field and electric potential simulations, appears to selectively engage the OFC (Figure 1). Our behavioral findings indicate that stimulation of this brain region reduces the devaluation of delayed rewards. This effect appears to be directly related to OFC stimulation, rather than mediated by other cognitive functions such as working memory. However, it is difficult to conclude that DD expression is solely mediated by the OFC since the stimulation effect size is limited.

In addition to processing the delay in the reward’s receipt, our brain has to weigh and evaluate the magnitude of rewards as well, a process that is likely to engage different neuronal circuits (Miyazaki et al., 2020). Although we observed an effect of maximal reward magnitude on the devaluation curves, related to the so-called “magnitude effect” (Sun and Potters, 2022), our results do not provide sufficient evidence to support the idea that the OFC is selectively engaged for either high or low rewards levels, since the interaction between stimulation and reward magnitude was not statistically significant in our analyses. Hence, further investigations, e.g., testing other reward orders of magnitude, are required.

We observed an unexpected finding about the correlation between WM and DD: not only we did not find a significant correlation between the two domains, but the difference in n-back performance between SHAM and Real stimulation conditions was not significant. This likely suggests the independence of the two processes, although in the literature the opposite is often suggested (Wesley and Bickel, 2014). Further investigation is required to clarify this point.

It is noteworthy that the medial OFC, which is believed to be involved in the corticostriatal pathway that encodes the subjective value of rewards (Fettes et al., 2017), was only marginally stimulated by our configuration. Therefore, future experiments should aim at targeting this region using more direct approaches such as transcranial magnetic stimulation to further explore its involvement in DD.

Since DD is regarded as a transdiagnostic process and many psychiatric patients exhibit greater impulsivity in intertemporal choice tasks (Frost and McNaughton, 2017), our results may have implications for clinical purposes. Addicted subjects, for example, who are more prone to choose the immediate options in decision tasks, and whose temporal devaluation is generally very high (Weinsztok et al., 2021), also display alterations in the activity of OFC (Volkow et al., 2004). Therefore, the tDCS configuration used in this study could be tested as an effective rehabilitation protocol for several psychiatric and psychopathological conditions, to ameliorate the maladaptive decision behaviors shown by these patients.

## Data availability statement

The original contributions presented in this study are included in the article/supplementary material, further inquiries can be directed to the corresponding authors.

## Ethics statement

The studies involving humans were approved by the Ethics Board of the Faculty of Psychotherapy Science and the Faculty of Psychology of Sigmund Freud University. The studies were conducted in accordance with the local legislation and institutional requirements. The participants provided their written informed consent to participate in this study.

## Author contributions

ASM, DS, MF, AV, and JL: conceptualized the experiments. ASM and DS: performed the experiments. ASM, DS, and JL: analyzed the data. ASM: writing–original draft preparation. ASM and JL: writing–review and editing. MF, SS, JL, and AM: supervision. All authors have read and agreed to the published version of the manuscript.

## Footnotes

1
www.jspsych.org

2
https://www.r-project.org/

3
https://github.com/andypotatohy/roast
